# Student’s Second-Language Grade May Depend on Classroom Listening Position

**DOI:** 10.1371/journal.pone.0156533

**Published:** 2016-06-15

**Authors:** Anders Hurtig, Patrik Sörqvist, Robert Ljung, Staffan Hygge, Jerker Rönnberg

**Affiliations:** 1 Department of Building, Energy and Environmental Engineering, University of Gävle, Gävle, Sweden; 2 Linnaeus Centre HEAD, Swedish Institute for Disability Research, Linköping University, Linköping, Sweden; 3 Department of Education, Health and Social Science, University of Dalarna, Falun, Sweden; 4 Department of Behavioral Sciences and Learning, Linköping University, Linköping, Sweden; Kyoto University, JAPAN

## Abstract

The purpose of this experiment was to explore whether listening positions (close or distant location from the sound source) in the classroom, and classroom reverberation, influence students’ score on a test for second-language (L2) listening comprehension (i.e., comprehension of English in Swedish speaking participants). The listening comprehension test administered was part of a standardized national test of English used in the Swedish school system. A total of 125 high school pupils, 15 years old, participated. Listening position was manipulated within subjects, classroom reverberation between subjects. The results showed that L2 listening comprehension decreased as distance from the sound source increased. The effect of reverberation was qualified by the participants’ baseline L2 proficiency. A shorter reverberation was beneficial to participants with high L2 proficiency, while the opposite pattern was found among the participants with low L2 proficiency. The results indicate that listening comprehension scores—and hence students’ grade in English—may depend on students’ classroom listening position.

## Introduction

The Swedish National Agency for Education—the central administrative authority for public school in Sweden—organizes nationwide tests on a yearly basis. The students’ test results are used for grading and selection purposes. One of the tests—that is part of a larger test battery that is supposed to measure second language proficiency—is *English listening comprehension*. In this test, the pupils listen to spoken conversations that are played back through loudspeakers, typically stationed at the front end of the classroom. This circumstance could lead to a situation wherein the pupils are not taking the test under equal premises, which could have unfair consequences for grades based on task scores. Room acoustic factors may differ between classrooms wherein the test is administered, and the pupils’ position in the classroom, in particular the distance to the loudspeaker, may influence test scores. The purpose of the present study was to investigate the interaction between classroom acoustic factors (i.e., reverberation) and distance between listener and sound source (i.e., the listener’s position in the classroom, relative to an audible loudspeaker (in all conditions) at the front desk). Room acoustic factors, such as background noise and reverberation, influence speech perception and comprehension. For example, when reverberation time—i.e., the time it takes for an auditory signal to drop 60 dB after the sound source has been turned off—is prolonged, speech perception is compromised. Reflected sound that exceeds 35 msec from the direct sound makes it harder to comprehend the speech [1–2]. Moreover, reverberation typically impairs memory of spoken lectures [3] and memory of spoken word lists [4], even when the listening conditions are assumed to be acceptable according to prevailing acoustical norms [3]. However, there is a possibility that reverberation not always has a detrimental effect. We have previously found in our research that a long reverberation time can improve recall for words spoken in English and Swedish when compared with a short reverberation time, at least when the signal-to-noise ratio is low [5–6].

The effects of classroom reverberation on cognitive performance are, thus, complex. One complicating factor is that the effect of reverberation upon the speech signal that reaches the ear depend on the distance between the position of the sound source (e.g., the teacher speaking from the front desk) and the position of the listener (i.e., the pupil). The acoustic factors of the classroom will be more pronounced when the distance is large, as the sound to a greater extent will bounce against wall and ceiling surfaces before reaching the ear. In particular, children experience significant speech perception difficulties when seated in the middle to the rear end of the classroom [7–8]. The effects of noise/reverberation distort the speech signal whilst distance from the sound source influences sound pressure level. Hence, different mechanisms (e.g. cognitive and acoustical) underpin the effects of masking and distance on speech perception (the process by which the sounds of language are heard, interpreted and understood). A long reverberation time affects speech intelligibility [9–10] and distance affects sound pressure level [11]. However, listening positions near a speaker receives sound directly and just a small portion of reflected sound waves. Whereas, listeners seated at the end of the classroom get a larger proportion of the sound as reflected sound. Thus, the reverberation time in classrooms are perceived as more distorting with a greater distance between speaker and listener [11]. The two factors may not interact in their effects on cognition in view of this independency. However, the magnitude of room reverberation varies with listening position within a classroom [4], whereby the two factors may interact. The reverberation time of the room should have a relatively small effect on the quality of the speech signal that reaches the ear, when the distance between speaker and listener is short, whereas the effect should be relatively large when the distance is larger.

An internal factor—as opposed to external factors like room acoustics—that influences speech intelligibility is the listener’s language proficiency [9–11], especially when the spoken message is masked by noise [12], by background speech [13], or when the reverberation time is relatively long [14]. For instance, children with English as second language (L2) experience greater speech perception difficulty when listening to English in the presence of background noise and/or reverberation, than native English speaking children, [8, 15–18]. Because of this, we decided to measure baseline L2 proficiency in the present study to make sure that the two independent samples were roughly equal in this skill.

In sum, the present study had two aims. First, to investigate whether the distance between the listener (i.e., the pupil) and the sound source in a classroom (i.e. loudspeaker at the front desk) influences scores on national English (i.e. the listener’s L2) listening comprehension tests; secondly, to investigate whether distance interacts with classroom reverberation. To this end, a standardized national English listening comprehension test was administered and scored the same way as in sharp situations, and high school students were recruited as participants to obtain a subject pool that is representative for the population to which the results should be generalized. On the basis of previous research, it was predicted that there would be differences in listening comprehension based on classroom reverberation time and on the distance from listening position to the sound source and that these two factors interact.

## Materials and Methods

### Ethical approval

For this research we have a legal ethical approval from the Regional Ethical Board in Uppsala (Nr 338/2011), which allows us to make studies of pupils aged 15 years old or older without asking approval from parents or guardians. All of the participants in the present study were at least 15 years old, the school administration gave their consent to the study, and the pupils were given the option to opt out from the study, which no one did.

### Participants

All participants had to be native Swedish speakers, and they had to have no impairments in hearing or reading abilities, as revealed by self-reports. A total of 133 Swedish high school students (73 females), all 15 years old, took part in the experiment in exchange for cinema tickets. All were native Swedish speakers. Eight participants did not follow the instructions that were given and were therefore removed prior to the analysis.

### L2 listening comprehension test

Two different L2 listening comprehension tests, taken from the National Tests of English for senior Swedish high school students were administered. Each test involved listening to a conversation spoken in the listeners’ second language (*i*.*e*., English) by native English speakers, presented over headphones. The two conversations/sound files ranged from approximately 11 to 12 minutes in length. The standard administration procedure for the National Tests of English was used, with the exception that the sound was presented over headphones instead of using a loudspeaker to obtain experimental control of the variables that were under investigation. In other words, the regular administration procedure was simulated.

To simulate different room acoustic conditions, varying in reverberation time and varying in different classroom positions relative to the loudspeaker at the front desk, two different rooms and two different positions were designed in the sound simulation program CATT-Acoustic. The rooms were equal in shape, but differed in terms of reverberation time. The mean reverberation time (250 Hz to 4 kHz) for the two rooms were 0.33 sec, and 1.07 sec, all measured with T_30_ (measures the time for a sound level to drop by 30 dB). All sound files used in the present study were simulated in these two modeled rooms. In the long reverberation time classroom the sound source was positioned at the front of the room and the recording position was 6.13 meters from the sound source at the back of the room. In, the short reverberation time classroom the recording position was 1.05 meters from the sound source at the front of the room. The Speech Transmission Index (STI (a measure of speech intelligibility, ranging from 0–1, where 1 is a perfect speech transmission)) for the short reverberation time classroom recordings were 0.95 (short distance), and 0.84 (long distance) and 0.71 and 0.62 respectively for the long reverberation time classroom recordings.

### Baseline L2 proficiency test

An English reading comprehension test was used to measure baseline L2 proficiency. We used this English reading test to measure baseline L2 because it was easier to distribute and because of the correlation between reading and listening comprehension [19–22]. As with the L2 listening comprehension test, this reading comprehension test is part of the Swedish National tests of English proficiency in senior high school students. The reading comprehension test consisted of a text, presented on an A4-paper, and a separate paper with 8 comprehension questions for which the participants could obtain a maximum of 12 points. The participants had a maximum of 12 minutes to complete the task. They had the question sheet and the story material available during the full 12 minutes.

### Design and procedure

A Split Plot analysis of variance was used with two factors. One factor was listening position (*i*.*e*., the distance between sound source and listening position) and this factor was manipulated within participants and had two levels, 1.05 m versus 6.13 m between source and receiver. The other factor was classroom reverberation time, manipulated between participants and had two levels, 0.33 sec and 1.07 sec respectively. Each participant sat in front of a computer monitor in groups of 10–11 participants at a time. The participants first undertook the baseline L2 proficiency test, followed by the two L2 listening comprehension tests (in counterbalanced order between participants).

## Results

The two independent samples, the group who took the listening comprehension tests in the classroom with the long reverberation time and the group who took the test in the classroom with short reverberation time, had comparable scores on the baseline L2 proficiency reading test (*M*_long_ = 7.5, *SD* = 2.6, *N* = 64; *M*_short_ = 8.0, *SD* = 3.1, *N* = 61), and the scores did not differ significantly, *t*(123) = 1.00, *p* = .328. Hence, the comparison between the two groups was arguably not influenced by differences in baseline L2 reading proficiency. A split close to the median of the L2 reading score into the new dichotomized variable Readgroup (low ≤ 8.0, *N* = 63; high > 8.0, *N* = 62) was employed as a between-person variable in our analyses. Thus, the ensuing ANOVA had Position in the classroom (close-far) as a within-person factor and Readgroup (low-high) and Reverberation time (short-long) as between-person factors.

**Fig 1:**
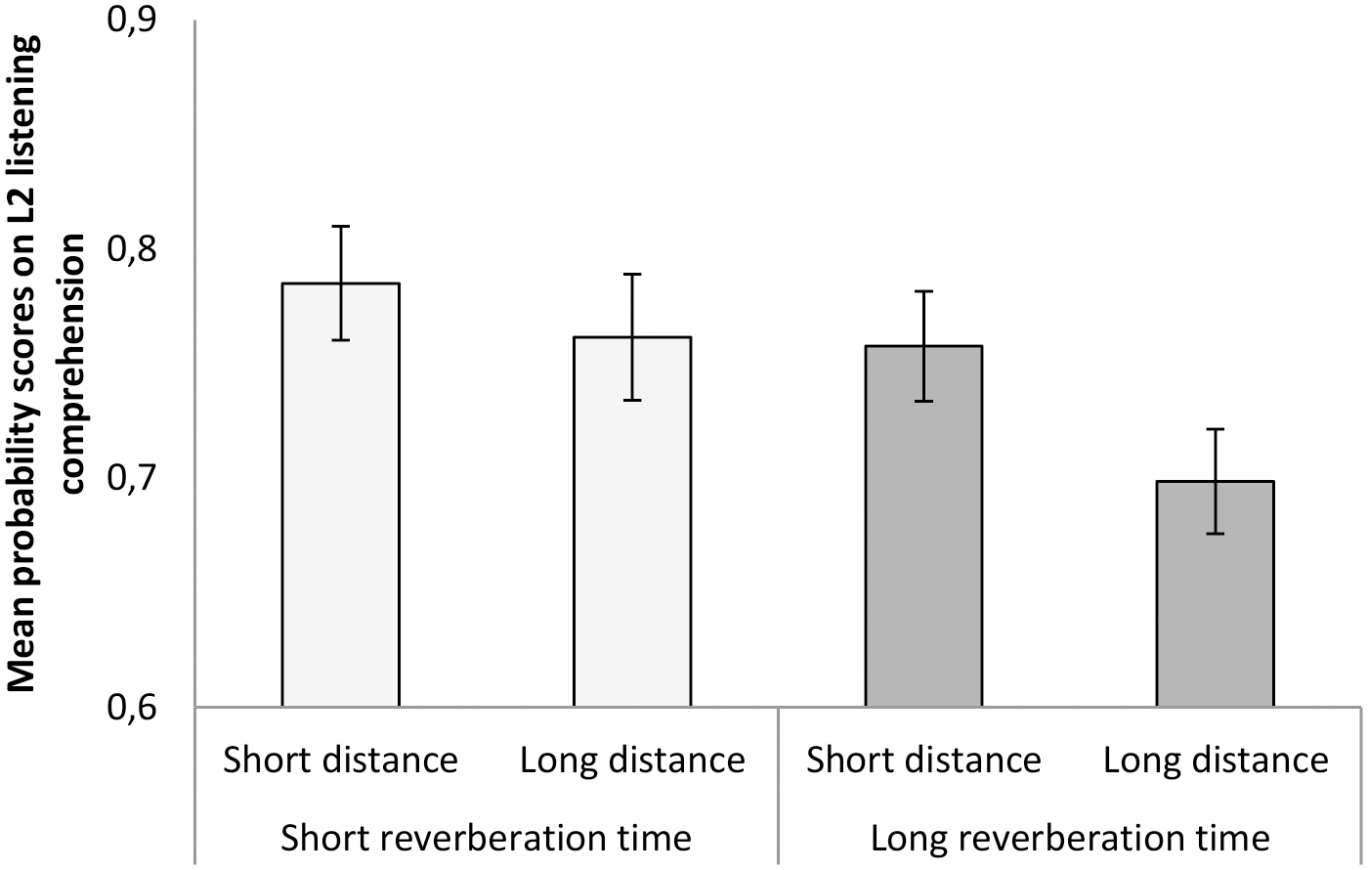
Mean probability scores on L2 listening comprehension. Swedish speaking participants’ scores on a second-language (English) listening comprehension test (as part of the National Tests of English in the Swedish School System) in a classroom with a short reverberation time (0.33 sec) and a classroom with long reverberation time (1.07 sec) at two different distances from the sound source (1.05 m versus 6.13 m away from the sound source). Error bars represent standard error of means.

**Fig 2:**
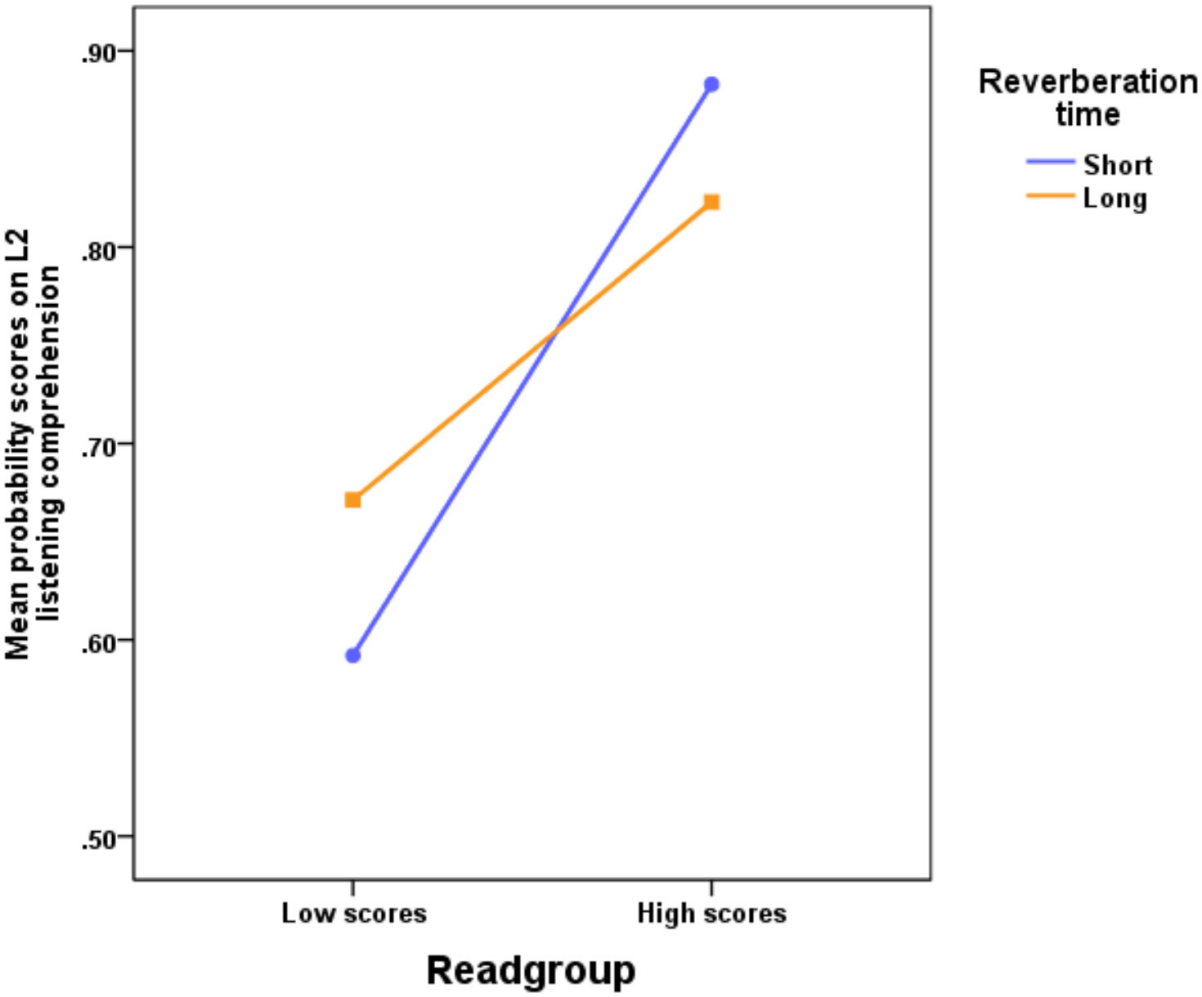
Mean probability scores on L2 listening comprehension for participants with different second-language abilities. Interaction between Swedish speaking participants’ scores on a second-language (English) reading comprehension test (readgroup divided in low and high scores, split by the median) in a classroom with a short reverberation time (0.33 sec) and a classroom with long reverberation time (1.07 sec).

As indicated in Fig 1, the participants obtained lower scores on the L2 listening comprehension test when there was a greater distance between the participants and the sound source, in comparison to when the distance was shorter, *F*(1, 121) = 4.41, *p* = .038, η_p_^2^ = .035. Thus, when the 15-year-old participants’ were seated more than 6 meters away from the sound source (with an STI = .62) there was a lower test score than when the participants sat 1.05 m away from the sound source (STI = .71). The magnitude of this Position effect was similar in the two reverberation time conditions as indicated by the lack of significant interaction between reverberation time and distance to sound source, *F*(1, 121) = 0.579, *p* = .448, η_p_^2^ = .005. Further, there were significant effects of Readgroup, *F*(1, 121) = 40.28, *p* < .001, η_p_^2^ = .250, and the interaction Reverberation time*Readgroup, *F*(1, 121) = 3.98, *p* = .048, η_p_^2^ = .032, as shown in Fig 2. The three-way interaction was non-significant, as was no other interaction, besides Reverberation Time*Readgroup. A closer inspection of the interaction indicates that the 95% CIs of the difference scores at Readgroup Low (= .079) and High (= .060) mutually exclude each other. Thus, the pupils who were good at reading English benefited significantly from a short reverberation time, while those who were not so good at reading English gained more benefit from a long reverberation time. Besides our previous research [5–6], we have not encountered any other research that has found an enhancement effect on recall or speech comprehension from a long reverberation time. All previous research on the effects of reverberation on recall and speech comprehension has only shown detrimental effects.

## Discussion

The major finding was that L2 listening comprehension decreased as distance from the sound source increased. This result is not likely due to difficulties hearing what was played back since all participants reported normal hearing. Some of the participants scored the highest possible result on the listening test in the worst condition (longest distance from sound source and longest reverberation time) confirming that it was possible to hear all that were said even in this condition. The distance between sound source and receiver did not interact with classroom reverberation. This finding was a new and surprising finding. It appears that a position further away from a sound source in a classroom has a higher detrimental effect on recall than a long reverberation time (1.07 sec). The results accord with findings by Crandell [7] and Crandell and Smaldino [8], who both showed that student’s position in a classroom can have detrimental effects on listening comprehension. This position factor on L2 listening comprehension is significant even for 15-year-olds, as we have shown here. When seated at a greater distance from a sound source (in this case a loud speaker stationed at the front of the classroom) the sound pressure level is lower in the back row than it is in the front row. Because STI is lower when the distance between the sound source and the receiver is large, one must challenge the auditory system more than when STI is higher, leaving fewer cognitive resources left to complete listening comprehension.

Taken together, the position effect appears to be quite robust to classroom reverberation, at least within the limits of the manipulation used here (i.e., two reverberation conditions that are representative of realistic classrooms, 0.33 sec and 1.07 sec), but the combination of a long reverberation time and a greater distance from the sound source may still be the most adverse condition for L2 test taking as indicated by Fig 1, even though there was no significant interaction between the two.

There was, however, an interaction between readgroup and classroom reverberation, where the low-score reading group scored relatively higher with a longer reverberation time than in the shorter reverberation time condition. In the high-score reading group the results were the opposite, scores were relatively higher in the shorter reverberation time condition compared to the longer reverberation time condition. These results are in line with our previous research [5–6]. The argument for this phenomenon is that with more time available (i.e. longer reverberation time) for processing words in first and L2 demands less resources and, hence, more resources are available for elaboration with encoding, storage and recall. A longer reverberation time could therefore assist L2 listening comprehension and recall for participants who score low on L2 reading comprehension. Conversely, when participants score high on a L2 reading comprehension test, a long reverberation time on an English listening test may only have a signal distorting effect. De Martino, Espesser, Rey, and Habib [23] reported that pathological language difficulties have their basis in a temporal processing deficit, and slower presentation times of the to-be-understood material can be particularly assisting in these populations. A possible hypothesis is that, when language perception processes are impaired, by internal person-specific cognitive abilities or by external environmental factors like position in a classroom, a long audible presentation time of the to-be-remembered material assist the temporal integration of the speech signal and, hence, makes it easier to understand and recall. This is speculative, but may be of interests for future research of a potential benefit from a longer reverberation time—at least within populations with underdeveloped language skills and with children [5–6].

The cross-over interaction between reverberation and readgroup found in this experiment shows that participants who score low on an English reading test, benefit from a longer reverberation time than their high scoring counterparts. A similar interaction was shown in previous research with reverberation and signal-to-noise ratio (SNR) [5]. In that experiment we found a cross-over interaction between SNR and reverberation time in grade 4 children (10 years old) where a high SNR (+12 dBA) and a short reverberation time (0.3 sec) improved recall, but impaired recall at +3 dBA. Those results resemble the results found in this experiment. This cross-over interaction doesn’t go in line with the assumption that noise (i.e. reverberation) in a classroom always has detrimental effects on recall. It could be argued that poorly achieving school children/adolescents’ perform better when reverberation is longer because they have more time to elaborate and assimilate the learning material.

It may be useful to acknowledge that the largest difference found here was that between the condition with short reverberation time and a shorter distance between sound source and receiver, and the condition with a long reverberation time and a further distance between sound source and receiver (Fig 1).

The Swedish National Agency for Education—the central administrative authority for public school in Sweden—organizes nationwide tests on a yearly basis. The results from these tests are used for grading and selection purposes. Therefore, it is crucial that all materials and all test procedures are standardized. Yet, as long as these tests are administered in dissimilar classroom environments, it is possible that this goal cannot be achieved. The results reported here could potentially suggest that this circumstance could lead to a situation wherein the students are, in fact, not taking the test under equal conditions.
